# Cutaneous Melioidosis in Adolescent Returning from Guadeloupe

**DOI:** 10.3201/eid1802.111603

**Published:** 2012-02

**Authors:** Roderich Meckenstock, Audrey Therby, Stephanie Marque-Juillet, Sebastian Monnier, David Khau, Beatrice Pangon, Alix Greder-Belan

**Affiliations:** Versailles Hospital, Le Chesnay, France

**Keywords:** melioidosis, Guadeloupe, Burkholderia pseudomallei, bacteria, primary cutaneous melioidosis, Caribbean, France

**To the Editor**: Melioidosis is an emerging zoonosis caused by a highly invasive and drug-resistant bacterium, *Burkholderia pseudomallei*, that is found in soil and is endemic to Southeast Asia and the Pacific region. Few cases occur in other regions (most imported by travelers) (*1**–**5*), but sporadic cases have originated from the Caribbean (*6**–**8*).

Melioidosis can manifest many years after exposure to *B. pseudomallei* and can cause severe, systemic infection, including multiple abscesses of internal organs and skin. A less severe manifestation, primary cutaneous melioidosis, causes skin lesions and milder clinical illness. We describe an adolescent patient who had a benign, cutaneous form of melioidosis; she had recently returned to France from Guadeloupe, a Caribbean archipelago.

A 15-year-old girl without a medical history, except for asthma, was evaluated in September 2010 for muscle weakness, weight loss of 15%, cough, and fever >40°C. During a trip to Guadeloupe 3 weeks before, she had been infected by dengue virus, along with her brother and father, who recovered rapidly. Treatment with amoxicillin and clavulanic acid was started after her return to France, despite the lack of a clear diagnosis, and induced a slight decrease in fever.

Clinical examination showed a body mass index <15, multiple small adenopathies (<10 mm), small papulous skin eruptions, and an inflammatory 15-mm–wide tumefaction on the upper arm, evoking an adenopathy on ultrasound investigation. Biological screening 2 weeks later showed persistence of inflammation. Results of serologic tests for cytomegalovirus, Epstein-Barr virus, parvovirus B19, chikungunya virus, *Rickettsia*, *Coxiella*, *Chlamydia*, *Brucella*, and *Borrelia* spp. did not show acute infectious disease; results were positive for recent mycoplasma infection, despite absence of typical signs and symptoms. A 2-week treatment regimen with spiramycin was started; general improvement followed, and the cough resolved.

The tumefaction of the upper arm persisted, and a biopsy was performed. Histologic results were nonspecific; culture on sheep’s blood Columbia agar and chocolate agar produced small colonies of gram-negative bacilli after 24 hours’ incubation at 35°C in an atmosphere of 5% CO_2_. This bacillus was later identified as *B. pseudomallei* by using the Vitek2 test card (bioMérieux, Marcy l’Etoile, France). Identification was confirmed by sequencing of 16S rRNA.

The patient was treated with intravenous ceftazidime (150 mg/kg for 10 d), followed by oral cotrimoxazole (800 mg of trimethoprim and 160 mg of sulfamethoxazole, 2×/d), with a total treatment duration of 12 weeks. Eleven weeks after treatment ended, the patient had recovered, and the tumefaction of the arm had disappeared.

The differential diagnosis for primary cutaneous melioidosis includes pyogenic abscesses, insect bites, mycobacterial and other granulomatous lesions, and adenopathies, but melioidosis is usually not suspected in these conditions, particularly in patients from non–disease-endemic regions such as the Caribbean. Clinical phenotypes of melioidosis range from asymptomatic carriage to fulminant shock syndrome (*1**–**5*); death rates for the latter are ≈100% (*3*). Subacute melioidosis may be associated with pulmonary and general signs; chronic variants could give rise to abscesses or septicemia in cases of concomitant immunodeficiency (*1**–**5*), even decades after exposure. Signs and symptoms of melioidosis can mimic those of tuberculosis, even though there is no link between the infectious agents (*2**,**4*).

Cutaneous melioidosis may be primary (a single, nonspecific, sometimes ulcerated lesion, measuring from several millimeters to several centimeters) or secondary (multiple lesions associated with visceral infection). In a study of 486 melioidosis patients in Australia, 58 (12%) had the primary cutaneous form (*9*). These cases were characterized by younger patient age (more common among children <16 years of age), higher frequency during the dry season, better prognosis in spite of a possible chronic evolution, and absence of classic risk factors (*9*) such as diabetes, alcoholism, chronic renal or pulmonary infections, surgery, pregnancy, or cystic fibrosis (*1**–**5*).

The case we describe is consistent with the cutaneous variant of melioidosis. However, the patient’s initial general symptoms (probably attenuated by early treatment with antimicrobial drugs) could have indicated a transitory, disseminated phase of disease such as that experienced by 4 (all adults) of the 58 cases of primary cutaneous melioidosis in the Australian study (*9*). It is not known whether *B. pseudomallei* was transmitted to the patient by an airborne route or percutaneously as in most cases (i.e., wounds infected by contaminated water or mud); other transmission modes are anecdotal (*1**–**5*). Moreover, our patient had none of the classic risk factors, although dengue fever as an underlying co-infection has been described (*10*).

The patient was treated with intravenous ceftazidime and oral cotrimoxazole at the minimum treatment duration recommended for melioidosis (*1**–**5*). Purely cutaneous variants of melioidosis may be treated exclusively by oral cotrimoxazole over 12 weeks (*9*), but we opted to prescribe initial intravenous treatment because of her general symptoms. We stopped follow-up 11 weeks after the treatment period ended because of persisting illness remission, but lifelong monitoring is recommended for adult patients (*1**,**4*) because relapses occur in ≈10% of adult patients despite well-conducted antimicrobial drug treatment (*3**,**4*).

In conclusion, melioidosis as a potential emerging infectious disease should be considered in cases of isolated skin lesions as well as in cases of unexplained fever with nonspecific symptoms. Furthermore, the disease should be considered not only among travelers returning from known disease-endemic regions but also in those coming from the Caribbean.
